# Synthetically defined glycoprotein vaccines: current status and future directions†This perspective is dedicated to the memory of Professor David Y. Gin.


**DOI:** 10.1039/c3sc50862e

**Published:** 2013-05-16

**Authors:** Roberto Adamo, Alberto Nilo, Bastien Castagner, Omar Boutureira, Francesco Berti, Gonçalo J. L. Bernardes

**Affiliations:** a Research Center , Novartis Vaccines and Diagnostics , Via Fiorentina 1 , 53100 Siena , Italy . Email: Roberto.Adamo@novartis.com; b Department of Chemistry and Applied Biosciences , ETH Zürich , Wolfgang-Pauli-Str. 10 , 8093 Zürich , Switzerland; c Departament de Química Analítica i Química Orgànica , Universitat Rovira i Virgili , C/Marcel·lí Domingo s/n , 43007 Tarragona , Spain; d Department of Chemistry , University of Cambridge , Lensfield Road , Cambridge , CB2 1EW , UK . Email: gb453@cam.ac.uk; e Instituto de Medicina Molecular , Faculdade de Medicina da Universidade de Lisboa , Av. Prof. Egas Moniz , 1649-028 Lisboa , Portugal . Email: gbernardes@fm.ul.pt

## Abstract

Primary examples in vaccine design have shown good levels of carbohydrate-specific antibody generation when raised using extracted or fully synthetic capsular polysaccharide glycans covalently coupled to a protein carrier. Herein, we cover recent clinical developments of carbohydrate-based vaccines and describe how novel cutting-edge methodology for the total synthesis of oligosaccharides and for the precise placement of carbohydrates at pre-determined sites within a protein may be used to further improve the safety and efficacy of glycovaccines.

## Abstract 2

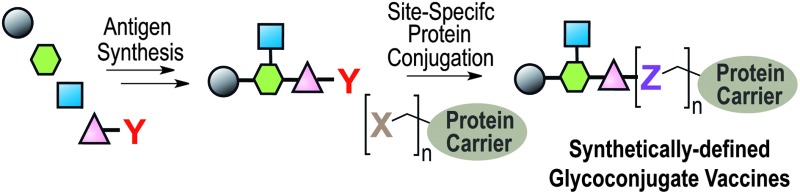
We highlight current glycovaccines in the clinic and derive principles for the construction of the next generation of synthetically defined glycoconjugate vaccines.

## 1 Introduction

Carbohydrate-based vaccines hold great promise for a number of diseases.^1^ The chemical nature of carbohydrate antigens presents a number of challenges with respect to inducing specific, protective antibodies: carbohydrates are poorly immunogenic, and in addition to that, carbohydrate-specific antibodies typically have low affinity (with *K*_D_ values in the micromolar range for monovalent interactions) compared with protein-specific antibodies (with *K*_D_ values in the nanomolar range). Thus, current strategies for the production of carbohydrate-based vaccines require conjugation of the low immunogenic carbohydrate antigen through a linker to a protein carrier for optimal antibody response. In this way, antibodies against both protein carrier and the less immunogenic glycan structures are generated enabling the targeting of carbohydrate antigens with vaccine strategies. Whilst the first evidence that conjugation of carbohydrate antigens to a suitable protein scaffold could enhance the immunogenicity of carbohydrates was reported back in 1931 by Avery and Goebel,^2^ it was only in 1987 that the first glycoconjugate vaccine was licensed for use in humans. The pioneering work of John Robbins' team^3^ that led to a glycoconjugate vaccine against *Haemophilus influenzae* type b is still considered a prototype for this class of vaccines. Since then, a number of successful glycoconjugate vaccines have been developed. Table 1 summarizes antibacterial glycoconjugates that are currently commercialized or are in advanced clinical trials, and details the carbohydrate source, the target infection and the conjugation chemistry used for their construction.

**Table 1:** Glycoconjugate vaccines licensed or in advanced development in the EU, US and WHO

Vaccine^*a*^	Target infection	Carbohydrate source	Conjugation chemistry	Development stage
*Manufacturer*				
PRP–TT	*Haemophilus influenzae* type b	Native polysaccharide	Carbodiimide-mediated condensation	Commercial
*Sanofi-Pasteur*				
PRP–OMPC		Medium length polysaccharide	Thioalkylation	
*Merck*				
PRP–CRM197		Native polysaccharide	Reductive amination	
*Pfizer*				
Hib–CRM197		Oligosaccharide from polysaccharide depolymerization	Active ester	
*Novartis V&D*				
PRP–TT		Synthetic oligosaccharide	Thiol-maleimide addition	
*CGEB, Cuba*				

MenC/Hib–TT	*Haemophilus influenzae* type b/*Neisseria meningitidis* serogroup C	Medium length polysaccharide (100–200 kDa)	Cyanylation	
*GSK*				

MenA–TT	*Neisseria meningitidis* serogroup A	Medium length polysaccharide (100–200 kDa)	Reductive amination	
*Serum Institute India*				

MenC–CRM197	*Neisseria meningitidis* serogroup C	Oligosaccharide from polysaccharide depolymerization	Reductive amination	
*Pfizer*				
MenC–CRM197		Oligosaccharide from polysaccharide depolymerization	Active ester	
*Novartis V&D*				
MenC-TT		Oligosaccharide from polysaccharide depolymerization	Reductive amination	
*Baxter*				

MenACWY–DT	*Neisseria meningitidis* serogroup A, C, W, Y	Oligosaccharide from polysaccharide depolymerization	Reductive amination	
*Sanofi-Pasteur*				
MenACWY–CRM197		Oligosaccharide from polysaccharide depolymerization	Active ester	
*Novartis V&D*				
MenACWY-TT		Medium length polysaccharide	Cyanylation/carbodiimide-mediated condensation	
*GSK*				

7 valent-CRM197 (4, 6B, 9V, 14, 18C, 19F, 23F)	*Streptococcus pneumoniae*	Native polysaccharide	Reductive amination	
*Pfizer*				
13 valent-CRM197 (1, 3, 4, 5, 6A, 6B, 7F, 9V, 14, 18C, 19A, 19F, 23F)		Native polysaccharide	Reductive amination	
*Pfizer*				
10 valent-DT/TT Protein D (1, 4, 5, 6B, 7F, 9V, 14, 18C, 19F, 23F)		Native polysaccharide	Isourea linkage/carbodiimide-mediated condensation	
*GSK*				
15 valent-CRM197 (1, 3, 4, 5, 6A, 6B, 7F, 9V, 14, 18C, 19A, 19F, 22F, 23F, 33F)		Native polysaccharide	Reductive amination	Clinical development (Phase II)
*Merck*				

3-valent-CRM197 (Ia, Ib, III)	Group B *Streptococcus*	Native polysaccharide	Reductive amination	Clinical development (Phase I)
*Novartis V&D*				

^*a*^CRM197: non-toxic mutant of diphtheria toxin; DT: diphtheria toxoid; OMPC: *Neisseria meningitidis* B outer membrane protein complex; Protein D: Hib related protein; PRP: polyribosylribitol phosphate; TT: tetanus toxoid.

Despite the central role of glycoconjugates in vaccination, there are few examples of synthetically defined glycovaccine candidates. Typically, even in cases where the glycan antigen is synthesised in a pure form, non-specific methods are used for the conjugation of the glycan antigens to the protein carrier. This results in mixtures of glycoforms with different glycan to protein ratios and glycosylation sites and, potentially, different pharmacokinetic and immunological properties. In this perspective we discuss the mechanistic principles underlying a specific and robust immune response; and how recently developed cutting-edge technologies in oligosaccharide synthesis and site-specific functionalization of proteins may help in designing and building chemically defined glycoproteins that enable a clear molecular dissection of vaccine structure on immune response and result in vaccine candidates with improved safety and efficacy.

## 2 Mechanistic considerations

Bacterial polysaccharides are high molecular weight molecules that, unlike proteins, are T-cell independent antigens and cannot be processed by antigen-presenting cells. They rather trigger B-cell responses by cross-linking the B-cell receptor, without any Major Histocompatibility Complex class II (MHCII) CD4^+^ T-cell interaction. An exception is represented by zwitterionic polysaccharides, such as pneumococcal serotype 1 polysaccharide or polysaccharide A from *Bacteroides fragilis*, which are capable of activating the adaptive immune system through processing by antigen-presenting cells and presentation by the MHCII pathway to CD4^+^ T-cells.^4^ As a result, bacterial polysaccharides induce B-cells to differentiate into plasma cells, leading to secretion of low affinity antibodies, predominantly IgM with some subsequent isotype switch to IgG, but without the formation of a persistent memory B-cell pool.

When polysaccharides are covalently linked to proteins, the resulting conjugates bind to the B-cell receptor of polysaccharide-specific pre-B cells and are taken into the endosomes. Once inside the cell, the protein portion is digested by proteases to release peptide epitopes, which are exposed on the surface in association with MHCII and presented to the αβ receptor of CD4^+^ T-cells. Peptide/MHCII-activated T-cells release cytokines to stimulate B-cell maturation to memory cells and induce immunoglobulin class switching from IgM to polysaccharide-specific IgG, so that upon exposure to the same carbohydrate antigen large amounts of high-affinity IgG antibodies can be produced. Consequently, immunization with glycoconjugates induces long-lasting protection against encapsulated bacteria, even among infants and persons in high-risk groups.

Recently, a new mode of action for carbohydrate-based vaccines has been proposed in which T-cells can recognize a pure carbohydrate epitope regardless of the peptide to which they are linked.^5^ The carbohydrate epitope bound to a peptide that results from endolysosomal processing of a Group B *Streptococcus* type III polysaccharide conjugated to a protein carrier, was shown to bind MHCII and to stimulate carbohydrate-specific CD4^+^ T-cell clones. While additional examples of carbohydrate-specific T-cell clones are needed to confirm the generality of these findings, the work reported by Avci *et al.* profoundly affects our understanding of the molecular mechanisms of antigen recognition by T-cells.

Hence, the general structure of a glycoconjugate vaccine consists of carbohydrate B-cell epitope and a protein or peptide providing the T-helper epitope to ensure T-dependent memory response. More complex constructs based on multivalent exposition of the haptens or incorporation of a covalently linked adjuvant have also been recently suggested and will be discussed below.

## 3 Glycoconjugate vaccine design

### 3.1 Choice of antigen

**Fig. 1:**
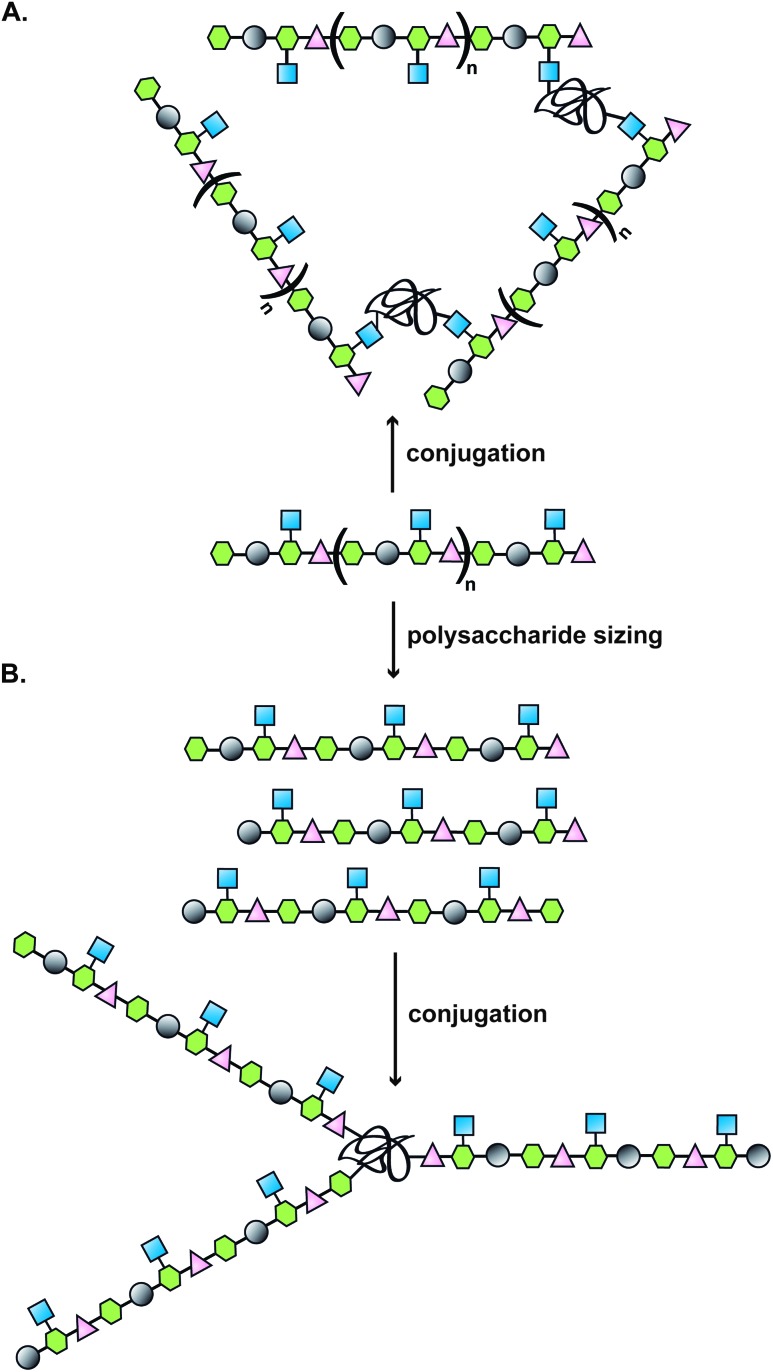
Illustration of a classical approach to the design of a carbohydrate-based vaccine using polysaccharides extracted from biological sources; (A) direct conjugation to carrier protein; (B) polysaccharide sizing followed by end terminal conjugation of generated oligosaccharides.

Typically, the route to a glycoconjugate vaccine commences with isolation and purification of the polysaccharide from a biological source (Fig. 1). Many bacteria, such as *S. pneumoniae*, *N. meningitidis* and *H. influenzae* produce a dense carbohydrate capsule, which represents an optimal natural supply of polysaccharides needed for eliciting specific antibodies able to confer protection against those bacteria.^1^ When a capsule is lacking, lipopolysaccharide can be sufficiently accessible by specific antibodies to be targeted in the development of a glycoconjugate vaccine, as demonstrated for *Vibrio cholerae* ^6^ or *Shigella dysenteriae*.^7^

In general, poly-/oligosaccharides from natural sources exist as heterogeneous mixtures of molecules with different degrees of polymerization. As immunogenic epitopes involved in the interaction with specific antibodies usually comprise precise glycan structures, often not longer than six or eight sugar units (45 year-old paradigm established by Kabat),^8^ significant efforts have recently been focused on the development of chemical and enzymatic methods for the synthesis of structurally well-defined carbohydrates with high purity. In rare cases even oligosaccharides as short as tetra- or even disaccharides have been shown to possess the minimal structural requirements for raising functional antibodies.^9,10^

**Fig. 2:**
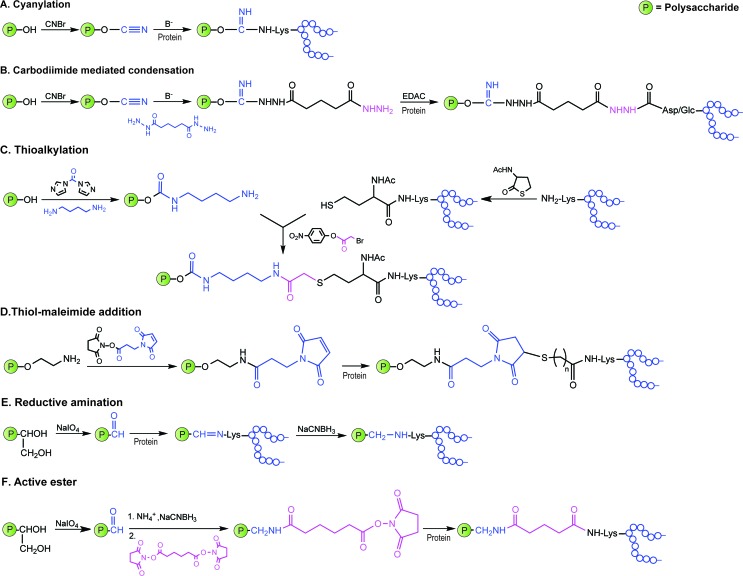
Illustration of commonly employed strategies for the conjugation of carbohydrate antigens (P = polysaccharide) to carrier proteins (protein) in licensed vaccines. These strategies often result in a mixture of glycoproteins due to the heterogeneity of the carbohydrate haptens and the variability of the attachment points onto the protein.

A breakthrough for synthetic vaccines was made in 2004 by a Cuban team who reported the large-scale synthesis and introduction of a synthetic oligosaccharide vaccine against *H. influenzae* type b in humans.^11^ Besides lacking bacterial impurities, synthetic oligosaccharides present the advantage of bearing at their reducing end a spacer amenable for chemoselective conjugation (see Fig. 2D), minimal batch-to-batch variability and higher quality control standards during process manufacturing, with respect to carbohydrates from natural sources.

Synthetic glycans from surface carbohydrates of diverse bacterial pathogens, including *S. pneumoniae* serotypes, *Shigella* species, Group A *Streptococcus*, *Staphylococcus aureus*, *V. cholerae*, fungal pathogens (such as *Candida albicans* and *Cryptococcus neoformans*)^12^ and the parasite *Plasmodium falciparum* ^13^ have been coupled to carrier protein and tested in animal models. Other emerging pathogens, such as *Clostridium difficile* ^14,15^ and *Francisella tularensis* ^16^ are being targeted using a similar approach.

Different variables can influence the immunogenicity of the glycoconjugate: some of them are correlated to the sugar hapten, such as chain length, non-reducing end residue (the more exposed sugar of the chain), charge, or presence of branching points; others depend on the conjugation chemistry and the type of spacer employed, which could direct the immunoresponse away from the sugar antigen, and the number of sugar moieties attached to the protein. All those parameters are case dependent and extremely important to identify the best carbohydrate antigens with correct exposition onto the protein, especially for short synthetic haptens.

Improved techniques for high-throughput glycan-binding assays, either based on covalent or noncovalent attachment protocols, are proving valuable tools to speed up identification of relevant glycan targets as vaccine candidates, by revealing the fine specificity of relevant carbohydrate-specific antibodies elicited during natural infection.^17^

### 3.2 Choice of protein carrier

Carrier proteins used for carbohydrate antigen conjugation are preferably proteins that are non-toxic, non-reactogenic and that can be obtained in sufficient amount and purity. They should also be stable under standard chemical conditions (concentration, pH, ionic strength) of conjugation procedures. Current proteins used in commercial or under development conjugate vaccines include: diphtheria toxoid (DT), nontoxic cross reactive material of diphtheria toxin (CRM_197_), tetanus toxoid (TT), keyhole limpet hemocyanin (KLH), the outer membrane protein of *N. meningitidis* (OMPC), recombinant exoprotein *Pseudomonas aeruginosa* (rEPA) and more recently protein D derived from non-typeable *H. influenzae*. In some cases, bacterial polysaccharides have been conjugated to a protein from the same pathogen to combine the effects of the carbohydrate and the protein antigens, as in the case of alginate–flagellin conjugates from *P. aeruginosa*.^18^

The choice of the carrier protein is particularly critical in designing cancer vaccines, due to poor immunogenicity of tumor-associated carbohydrate antigens (TACAs). Tumor cells are characterized by aberrant glycosylation patterns that result in heterogeneity, overexpression and truncation of surface oligosaccharides. Therefore, the concept that specific anti-carbohydrate antibodies can be utilized to boost the host's immune system to either treat (therapeutic vaccines) or to delay the relapse of a disease (prophylactic vaccines) can find application also in cancer. Three main categories of saccharide structures have been identified as potential TACAs: (1) mucin (MUC)-related *O*-glycans, including Tn (Gal-NAcα-*O*-Ser/Thr), TF (Galβ1-3GalNAcα-*O*-Ser/Thr), and STn (Neu5Acα2-6GalNAcα-*O*-Ser/Thr); (2) glycosphingolipids, including gangliosides GM2, GD2, GD3, fucosyl GM1, and neutral globoside globo H; and (3) blood-group antigens, including SLe^x^, Le^y^-Le^x^, SLe^a^, and Le^y^ in *N*-linked, *O*-linked, or lipid-linked structures.^19^ Isolation of these antigens from natural sources is a difficult task due to the heterogeneity of the cell-surface (for instance, globohexaosylceramide), thus the synthetic approach appears very attractive for the development of cancer vaccines. However, the induction of high-affinity IgG antibodies against TACAs has proven much more challenging than for saccharides derived from pathogens, because of their ‘self-antigen’ natures (they may be found in low concentration also on normal cells), and their presence in the blood-stream where they are shed by the growing tumor. Furthermore, pre-existing immunity to a carrier protein may suppress the immune response to the carbohydrate antigen linked to it.^20,21^ This phenomenon, known as carrier-induced epitope suppression, is particularly problematic when ‘self-antigens’ such as TACAs are employed for vaccine preparation.^19,22^

Studies on glycopeptides containing TACAs have pointed out that they are recognized by T-cells depending on the structure of the glycan moieties,^23^ demonstrating that it is possible to elicit specific MHC class II-restricted T-cell response against TACAs. In addition, after the recent discovery of Toll-like receptors (TLRs), which play a crucial role in the development of the adaptive immune response, it has become clear that their activation through specific adjuvants can control the release of cytokines and chemokines, which contribute to the interactions between T-helper cells and B- and antigen-presenting cells.^24^

All the aforementioned findings have greatly prompted the design of totally synthetic vaccines incorporating the carbohydrate antigen (B-cell epitope), a synthetic adjuvant and/or a selected universal helper T-cell peptide in place of the carrier protein with the aim of overcoming the immune-tolerance against TACAs.

Attachment of TACAs to lipopeptide Pam_3_Cys, which is a Toll-like receptor 2 (TLR2) ligand able to trigger the cytokine cascade activating dendritic cells, macrophages, and B-cells, evidenced that a small synthetic carbohydrate antigen can generate an immune response against the carbohydrate without a protein carrier, when a lipid emulsion is used for delivery.^25^ However the lack of a helper T-epitope precludes a class switch to IgG antibodies and affinity maturation, resulting mainly in the production of IgM antibodies.

The universal pan allelic HLA DR binding epitope (PADRE) peptide has been used for the synthesis of constructs containing unglycosylated, Tn (Gal-NAcα-*O*-Ser/Thr), and TF (Galβ1-3GalNAcα-*O*-Ser/Thr) modified MUC1, showing induction of IgG against the three B-cell epitopes.^26^ The same peptide or, alternatively, a CD4^+^ peptide T-helper epitope derived from polio virus were also coupled to trimeric Tn-antigen and mounted on a four arm lysine core to provide a multi-antigenic glycopeptide (MAG) which induced an immune response and promoted an increase in survival in tumor studies in mice, using both prophylactic and therapeutic settings.^27^ A fully synthetic three-component anticancer vaccine composed of the Tn-antigen, a helper T-epitope derived from *N. meningitidis*, and the TLR ligand Pam_3_Cys was initially designed and synthesized by Boons and co-workers and was shown to induce low to moderate IgG titers against the Tn-antigen in mice.^22^ In a subsequent study, two additional tri-component vaccine candidates composed of the tumor-related MUC1 glycopeptide, a helper T-cell epitope from polio virus, and either the TLR agonist Pam_2_CysSK_4_ or Pam_3_CysSK_4_ as adjuvants, were prepared and incorporated into liposomes. The vaccine bearing the Pam_2_CysSK_4_ adjuvant resulted in higher anti-MUC1 IgG antibody titers, and antibodies elicited by both constructs were shown to bind MUC1 expressing MCF7 tumor cells.^28^ More recently, αGalNAc glycosylation of MUC1 in a fully synthetic vaccine trimeric candidate composed of covalently linked immunoadjuvant Pam_3_CysSK_4_, a peptide T-helper epitope and an aberrantly glycosylated MUC1 peptide, in liposomal formulation, proved essential to induce more lytic antibodies toward tumor cells when compared to its unglycosylated counterpart, demonstrating that a tumor-specific anti-MUC1 response is attainable with an adequate set of vaccine components.^29^ The idea of utilizing synthetic peptides representing CD4^+^ T-cell epitopes has proven attractive also for antimicrobial vaccines, and a fully synthetic candidate combining β-mannan and the Fab peptide epitope has been demonstrated to induce protection against candidiasis.^30^

Novel platforms combining the multivalent approach and peptide carriers have also been exploited. A pentadecasaccharide from the lipopolysaccharide of *Shigella flexneri* and a Th peptide from influenza hemagglutinin (H307-325) conjugated to liposomes containing the immunopotentiator Pam_3_CAG induced IgM and IgG titers against the native lipopolysaccharide.^31^ Gold nanoparticles presenting a synthetic tetrasaccharide epitope related to pneumococcal type 14 polysaccharide, the T-helper ovalbumin 323–339 peptide (OVA323–339), and d-glucose elicited anti-polysaccharide IgG antibodies, albeit with lower bactericidal activities than the carbohydrate antigens conjugated to classic carrier proteins.^32^ These novel types of glycoconjugates are promising but require further developments including the use of promiscuous T-cell peptides able to interact with different MHCII in order to overcome their genetic variability in humans.

### 3.3 Choice of conjugation methodology

Covalent linkage of polysaccharides to proteins is generally achieved by targeting the amines of lysines, the carboxylic groups of aspartic/glutamic acids or the sulfhydryls of cysteines. Diverse approaches for the conjugation of carbohydrate antigens to proteins have been undertaken and are summarised in Fig. 2.^33^ Cyanate esters randomly formed from sugar hydroxyls can be reacted with the lysines of the protein or the hydrazine of a spacer which are then condensed to the carboxylic acids of the carrier protein *via* carbodiimide chemistry (Fig. 2A and B). Alternatively, aldehydes generated on purified polysaccharide by random periodate oxidation can either be directly used for reductive amination onto the amines of the carrier protein, or converted into amines for following insertion of a spacer enabling the conjugation step to the protein *via* thioether or amide bond formation (Fig. 2E and F). Glycoconjugates obtained by these methods present complex cross-linked structures (Fig. 1A). A strategy aimed at simplifying the structure of the final conjugate employs partial hydrolysis of the purified capsular polysaccharide and following fractionation to select an intermediate chain length population (Fig. 1B). A primary amino group can then be introduced at the oligosaccharide reducing termini to be used finally for insertion of either a diester or a bifunctional linker ready for conjugation to the protein.

These non-specific methods result in mixtures of glycoproteins with variable antigen loading number and antigen positioning. In Section 4.2 we present novel methodology that enables the precise glycosylation of proteins and that may be used to improve the safety and efficacy of glycoprotein vaccine candidates.

### 3.4 The need for multivalency

Nature frequently uses multivalency to achieve strong binding in situations where monovalent protein–ligand binding is weak.^34^ It is known that when carbohydrates are covalently linked to other biomolecules such as proteins or lipids, resulting avidities and selectivities of specific interactions are very dependent on the density of the sugar groups and also special organization of the scaffold to which the glycans are linked. In fact, in glycoconjugate vaccines, the length of the polysaccharide (which results in the repetition of minimal immunogenic structures along the polymer),^35^ and the degree of carbohydrate loading (number of conjugated glycans)^36^ are interdependent relevant variables, which influence the immunogenicity of the vaccine. Multivalent presentation of glycans has been applied to the design of glycopolymers, glycoclusters, glycodendrimers and glyconanoparticles which have been shown to interfere with pathogen adhesion, holding great potential to improve or replace antibiotic treatments that are now subverted by resistance.^37^

This concept has concomitantly aided the design of vaccine candidates attempting to target with specific anti-carbohydrate antibodies the dense ‘glycan shield’ that coats the surface envelope of the glycoprotein gp120 from the highly virulent human immunodeficiency virus (HIV).^1^ HIV viruses have evolved several defence mechanisms to escape the immune surveillance of the host immune system. Conformational rearrangements of the more exposed glycoprotein epitopes prevent the binding of specific antibodies, representing an obstacle to the success of a carbohydrate-based vaccine against this virus. After isolation of the broadly neutralizing monoclonal antibody (mAb) 2G12 (IgG) from one HIV-infected individual, highly conserved high-mannose-type *N*-glycan clusters on the gp120 surface were identified as possible targets for the development of an anti-HIV vaccine. In particular, Manα-(1,2)-Man moieties on the D1 and D3 arms of such *N*-glycans were found to be involved in binding the mAb 2G12. Furthermore, 2G12 exhibits a unique swapped VH domain structure, which generates a multivalent antigen binding surface with unusual affinity to the glycoprotein epitopes exhibiting a nanomolar dissociation constant.^38^ Early studies with the ultimate goal of eliciting “2G12-like” antibodies made clear that the design of the immunogens needed to rely on a multivalent presentation of chemically or chemo-enzymatically synthesized oligomannose clusters coupled to appropriate scaffolds to be further conjugated to protein carriers. Man_9_GlcNAc_2_ and Man_4_ could be identified as two favorable ligands for 2G12, and several strategies aimed at mimicking the high density of gp120 carbohydrate surface have been developed using bacteriophage Qβ,^39^ cyclic peptides^40^ and dendrimers^41^ as scaffolds. Recently, Davis and co-workers proposed a non-self sugar mimic for the design of novel ligands.^42^ Although in some cases antibodies could be raised against the oligomannose haptens, none of the constructs induced gp120 cross-reactive antibodies.

A step forward was made by using the yeast *Saccharomyces cerevisiae* glycoengineered to express Man_8_GlcNAc_2_ clusters on their surfaces.^43^ While gp120 cross-reactive mannose-specific antibodies were elicited in one rabbit by the yeast mutant glycoprotein, these antibodies did not neutralize HIV. Further crystallization studies are now better delineating the interaction between newly isolated potent neutralizing antibodies (PGT, PG9) and variable regions of gp120, which are critical for viral evasion of antibody neutralization.^44,45^ The involvement of two conserved glycans as well as a short peptide strand of the protein envelope for antibody penetration has been shown. These recent findings on the structural requirements needed for neutralization can fuel the development of novel immunogens against HIV.

The multivalent architecture of constructs bearing sugar antigens has also been exploited to enhance the immunogenicity of glycoconjugate vaccines targeting cancer.

Initial works on GD3-based conjugates established keyhole limpet hemocyanin (KLH) and QS-21 as the most potent carrier and adjuvant combination for raising antibodies against TACAs.^46^ After a preclinical study with globo H-KLH conjugate formulated with QS-21 showing complement-mediated lysis of tumor cell lines expressing the targeted TACA, a subsequent Phase I clinical study in metastatic breast cancer patients laid the groundwork for further clinical trials.^47^ A *N*-glycolyl (NGc) GM3-based vaccine obtained from the conjugation of the ganglioside into small size proteoliposomes (VSSP) derived from *N. meningitidis* is currently being tested in Phase III clinical trials in advanced melanoma and breast cancer patients showing acceptable safety outcomes and the ability to induce specific humoral and cellular immune responses.^48^

In order to more closely mimic the architecture of a tumor cell surface, a second generation of vaccines was designed using the concept of multivalency with trimeric clusters of one carbohydrate antigen (Tn, Tf, STn, 2,6STF or Le^y^). The constructs were shown to induce a more robust immune response with respect to their monomeric congeners, and some will soon enter clinical studies.^49,50^ Subsequently, a broader combination of sugar antigens associated with one particular cancer type was tested, and a hexavalent vaccine including GM2, globo H, Le^y^, glycosylated MUC1-32mer and clusters of Tn and TF conjugated to KLH and mixed with QS-21 induced good antibody titers against all the six haptens in high risk prostate cancer clinical patients during a Phase II study.^51^ The latter approach, albeit promising, necessitates the use of increased doses of carrier proteins, which can lead to reduction of the immune response against the carbohydrate antigens. Intensive efforts have been made to develop protocols for the synthesis of so called ‘unimolecular multivalent vaccines’, wherein several different carbohydrate antigens are displayed on a single polypeptide backbone, which requires conjugation to a single molecule of carrier protein. A pentavalent construct-KLH conjugate, incorporating Globo-H, GM2, STn, TF and Tn, showed high promise in inducing IgG and IgM antibodies against each of the five individual carbohydrate antigens, and is expected to enter Phase I clinical study.^52^

While these are important examples of advances in vaccine design and construction, optimization of the glycoconjugate multivalent constructs as well as their formulation is still required to achieve an efficacious anticancer vaccine.

## 4 Novel technologies for vaccine design

### 4.1 Oligosaccharide synthesis

Carbohydrate chemistry has only recently begun to impact the field of biology the way peptide and nucleic acid chemistry have. The challenges associated with carbohydrate chemistry are in part responsible for this discrepancy. Indeed, the assembly of oligosaccharides involves issues of regioselectivity, stereoselectivity, and branching that are not present for the other two biopolymers. Nonetheless, carbohydrate chemistry allows more than ever the synthesis of complex oligosaccharides. The incentive to synthesize carbohydrate antigen as opposed to isolation is manifold.^1,12^ Beyond their well-defined nature and high purity, synthetic antigens can help identify the best epitope and optimize its length in a way that would be difficult with isolated carbohydrates.^53,54^ Synthesis can also yield chemically modified epitopes that have improved immunogenicity because they break tolerance to self-antigen.^55,56^ The past two decades have witnessed the development of new methods allowing the once laborious syntheses to be performed with unprecedented efficiency.^17,57^ Some recent examples are presented below, with an emphasis on the developments that we believe have implications for the advancement of synthetic vaccines.

The assembly of oligosaccharides by chemical means requires monosaccharide building blocks that display carefully chosen protecting groups and an anomeric leaving group. The mammalian glycome is composed of a limited number of monosaccharides and linkages, such that a unified synthesis strategy could assemble a large proportion of known oligosaccharides using a limited number of building blocks.^58^ This will also be true for viruses, which make use of the host glycan machinery. Bacteria, on the other hand, have a much more diverse glycome, that could only be assembled using a correspondingly larger number of building blocks.^59^ In spite of that, the antigen “glycospace” within species will be more limited and tractable by synthesis. Recent progress has made the synthesis of protected building blocks to be used in oligosaccharide assembly more efficient. For example, a one-pot method to regioselectively protect monosaccharides dramatically cuts the number of steps necessary to obtain building blocks.^60^ While this method allows more efficient elaboration of building blocks from the corresponding monosaccharides, some building blocks can also be obtained efficiently by *de novo* chemical or chemoenzymatic syntheses.^61^ Indeed, whole oligosaccharides can be synthesized *de novo*, as elegantly demonstrated by the synthesis of a *Bacillus anthracis* exosporium tetrasaccharide.^62^

**Fig. 3:**
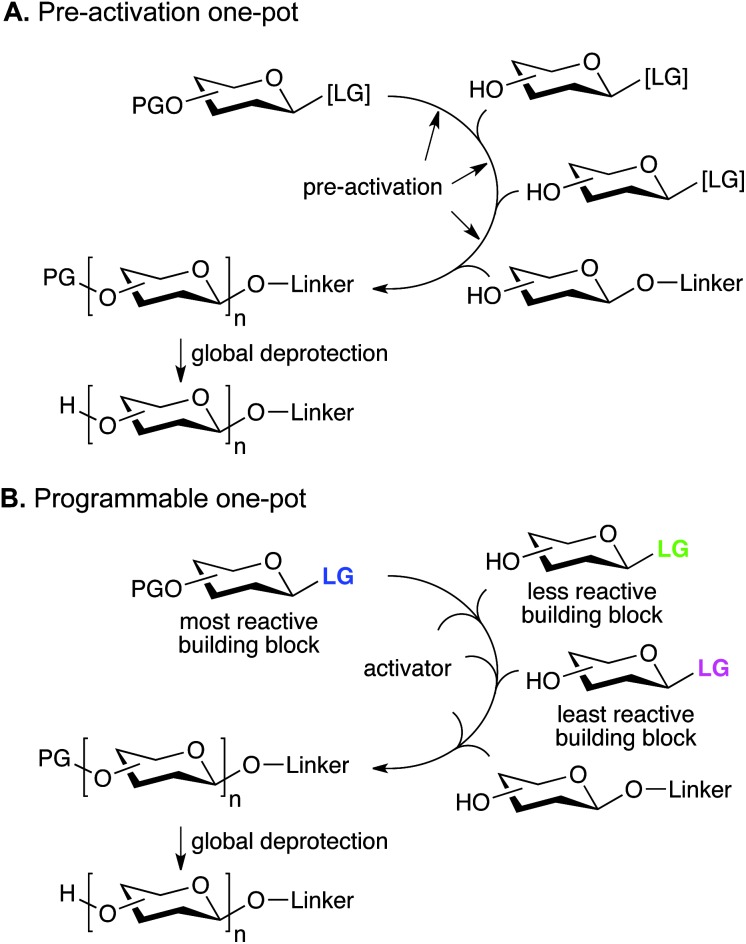
One-pot oligosaccharide assembly strategies (PG: protecting group, LG: leaving group, [LG]: latent leaving group).

Classical oligosaccharide assembly involves repetitive glycosylation and deprotection steps with potentially problematic purifications at each step. Combining multiple glycosylations in a so-called “one-pot” sequence dramatically cuts down the number of operations that have to be performed to obtain the desired oligosaccharide.^63^ Control over the sequence can be obtained by different strategies. In the pre-activation one-pot method, a latent leaving group can be pre-activated before glycosylation with a second building block, which contains both a free hydroxyl group and a latent leaving group (Fig. 3A). After the glycosylation, the latent leaving group of the newly incorporated building block is itself pre-activated before adding the next building block. Alternatively, in the reactivity-based one-pot strategy, a reactive building block can glycosylate a second, less reactive building block, containing a free hydroxyl group, under mild activation conditions (Fig. 3B). After the glycosylation, the newly incorporated building block can glycosylate the least reactive building block under conditions that will only activate the former. Then, the least reactive building block can be activated under forceful conditions to glycosylate the reducing end saccharide. In this method, the building block reactivity has to be tailored, usually by manipulation of the protecting or leaving groups. The one-pot sequence itself can be automated, as demonstrated in the parallel synthesis of a small library of oligosaccharides based on dimeric Le^x^.^64^ A disadvantage of the one-pot method is that different building blocks will be necessary depending of the precise structure sought. Therefore, it is not possible to imagine a common set of efficient building blocks that could form the basis for the synthesis of a myriad of structures. Finally, since only a few glycosylations can be performed in a one-pot sequence, di- and trisaccharide building blocks have to be used to assemble larger oligosaccharides.

**Fig. 4:**
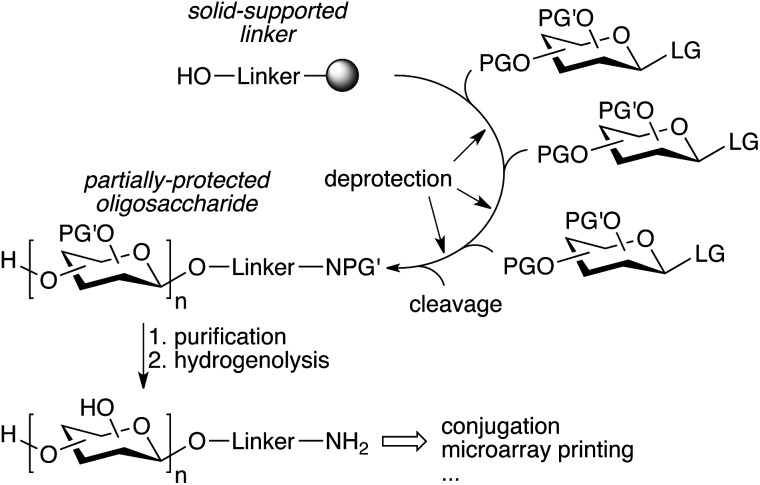
Streamlined automated solid-phase oligosaccharide synthesis (PG′: protecting group labile to hydrogenolysis).^69^

The repetitive nature of the oligosaccharide assembly is also well suited for solid-phase synthesis,^65^ similar to peptide and oligonucleotide synthesis, and the process has also been automated using a modified peptide synthesizer,^66^ and more recently an adapted HPLC.^67^ The strategy is based on a solid-supported linker bearing a hydroxyl that is glycosylated, followed by deprotection of the sugar hydroxyl that needs to be glycosylated (Fig. 4). The process is iterated until the desired oligosaccharide is obtained. A number of complex oligosaccharides have been obtained using this strategy.^68^ However, the modified peptide synthesizer was not adapted to the broader chemical and temperature range needed to perform carbohydrate chemistry compared to peptide coupling. Additionally, an olefin present in the linker prevented the use of thioglycosides and the final deprotection of the oligosaccharide after assembly was still laborious and challenging. In an effort to further streamline the synthesis process, a new automated platform including a new linker, a new custom-made synthesizer, and an improved chemical strategy was recently published (Fig. 4).^69^ The versatility of the new platform was demonstrated by the synthesis of a number of complex oligosaccharides ranging from sialosides, to the core *N*-glycan pentasaccharide and arabinofuranosides using a variety of building blocks, including thioglycosides.^69–71^ Importantly, the partially deprotected glycans obtained from the synthesizer could be fully deprotected using simple hydrogenolysis, minimizing manipulations after automated assembly.

These glycans all have an anomeric linker bearing a primary amine, allowing conjugation to proteins, glycan microarrays, *etc.* This is a significant step towards a more routine synthesis of complex glycans, as opposed to dedicated efforts by specialized laboratories. This strategy uses an excess of building block to ensure complete reaction, even for more challenging glycosylations. This is crucial to avoid lengthy optimizations before each oligosaccharide synthesis. Thus the broad utilization of this platform is intimately linked to the identification of a common set of building blocks and their efficient synthesis on a large scale. Unlike solid-phase synthesis, solution-phase synthesis potentially requires less excess of the building block to drive the reaction to completion. Therefore, soluble supports have been sought, such as fluorous-phase linkers.^72,73^ However, their progression to a fully automated platform has been challenging.^74^ Ultimately, the growing size of the oligosaccharide hinders its simple fluorous-phase extraction, and the technical complexity of the manipulations needed for synthesis and isolation complicates its automation.

The stereochemistry of the forming anomeric bond during glycosylation is still a challenge for carbohydrate chemistry. Valuable empirical data and systematic studies performed over the years now generally allow good control in most glycosylations. Nevertheless, the bacterial and mammalian glycomes include many “difficult” anomeric linkages, including 1,2-*cis* linkages, which elude traditional stereoselective methods. Fortunately new paradigms continue to emerge, such as the strategy involving a C2 protecting group bearing a sulfur atom, resulting in a well-defined rigid chair intermediate that forces the incoming nucleophile to attack from the α-face.^75^ Importantly, this method was amenable to solid-phase synthesis.^57^ β-Linked mannose is an important linkage, being present in the *N*-glycan conserved core. The intermediacy of a conformationally locked mannoside anomeric triflate at cold temperature allows selectivity in the formation of β-mannosides, which is also amenable to solid-phase,^76^ as well as automation.^77,78^ Sialic acid and related nonuloses also pose significant challenges to chemical synthesis due to the poor stereoselectivity and competing elimination of the activated building blocks. The synthesis of sialosides by one-pot methods or automated synthesis has largely relied on pre-formed building blocks.^69,71^ However, the group of Takahashi has reported a one-pot sequence incorporating sialic acids, including α-2,9-linked trisialic acid, using building blocks bearing different nitrogen protecting groups.^79^ The development of improved glycosylation protocols for complicated linkages will be instrumental to access a wide spectrum of antigenic glycans.

In contrast to the chemical approaches mentioned above, chemoenzymatic synthesis has the advantage of being exquisitely regio- and stereoselective, even in the absence of protecting groups.^17,80^ For instance, the chemically challenging sialic acid has been very efficiently incorporated into small glycans.^81^ The method uses optimised multi-enzyme systems that generate the sugar nucleotide *in situ*, and even the sialic acid from the mannosamine precursor. Furthermore, these examples demonstrate that while the range of chemical modifications possible is limited, unnatural analogs can be accepted as substrates in chemoenzymatic reactions. Chemical and chemoenzymatic synthesis are not mutually exclusive, and the latter can be used to elaborate oligosaccharides elaborated by chemical synthesis.

Owing to these significant advances, the synthesis front line now goes beyond carbohydrate assembly and into the presentation of well-defined scaffolds and the synthesis of ever more complex glycopeptide epitopes.^30,63,82^ The new challenges ahead not only reside in the synthesis, but also in the design of unnatural scaffolds and conjugates with enhanced functions, which can only be guided by the collaboration between chemists and vaccinologists.

### 4.2 Site-specific glycosylation of proteins

The ability to modify, manipulate and incorporate in a site-specific way glycosidic residues on proteins is widely accepted as one of the most important challenges in glycoscience. Glycosylation of biomolecules by the natural cell machinery is a prevalent post-translational modification (PTM) that results in complex mixtures of constructs with different glycosylation patterns (glycoforms).^83^ Such glycoforms are difficult to purify and characterize and different glycoforms can have different biological functions. In addition, incorporation of carbohydrates on proteins at pre-determined sites using the genetic machinery is still a rather difficult task.^84^ Chemical site-specific glycosylation methods can resolve availability and supply issues of homogeneous glycoprotein vaccine candidates. When combined with synthetically pure carbohydrate antigens (Fig. 3 and 4), site-specific strategies allow for the construction of synthetically defined glycoproteins with a precisely controlled epitope density and presentation. Site-specific chemical and (chemo)enzymatic transformations should be highly efficient and fast under mild conditions and operationally simple to ensure purity and homogeneity of the glycoproteins.

**Fig. 5:**
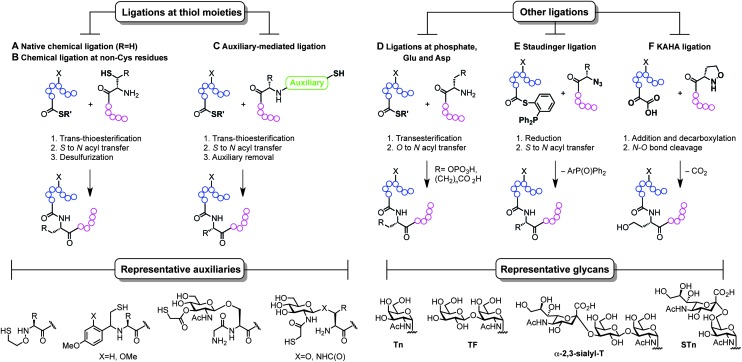
Total synthesis of (glyco)proteins using chemical ligation strategies.

**Fig. 6:**
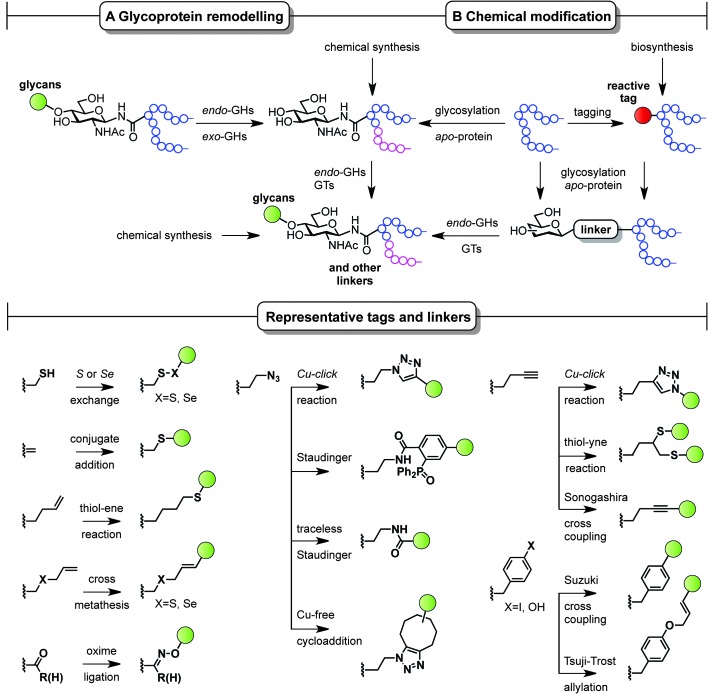
Chemoenzymatic (remodelling) and chemical strategies for the *in vitro* production of homogeneous glycoproteins. GHs = glycoside hydrolases and GTs = glycosyltransferases.

Classical non-specific glycosylation protocols exploit nucleophilic residues such as lysine and cysteine (Fig. 2). Under these conditions, mixtures of glycoproteins carrying a variable number of sugar epitopes at different sites are typically obtained. More recently, chemical strategies for the construction of well-defined glycoproteins have emerged and can be divided into three different general categories (Fig. 5 and 6). The first employs chemical ligation strategies (Fig. 5A–F) while the second involves chemoenzymatic transformations (glycoprotein remodelling) with endoglycosidases or other glycan processing enzymes (Fig. 6A). Finally, the last approach utilizes an organic synthesis toolbox adapted to work under biological conditions (aqueous media, mild pH and temperature), targeting and modifying both natural and unnatural amino acid residues (Fig. 6B).

The total synthesis of proteins, and particularly challenging glycoproteins, has attracted the efforts of the scientific community for more than 20 years, since Kent and Schnolzer developed a fully synthetic HIV-1 protease and a human interleukin 8 using chemical ligation^85^ (thioester as an amide surrogate) and native chemical ligation^86^ (NCL) (native amide bond with a cysteine at the ligation site) protocols, respectively. Unlike traditional strategies based on automated solid phase peptide synthesis that usually require long reaction sequences and protecting group manipulations in a linear synthesis scheme, the development of convergent total synthesis protocols that employ unprotected (glyco)peptide fragments represents a critical breakthrough for the preparation of glycoconjugates.^87^ The initial reports based on ligations at cysteine (Fig. 5A) inspired the development of alternative techniques where other thiol-containing residues were used (Fig. 5B and C). The overall transformation occurs in a three-step sequence: (i) *trans*-thioesterification, (ii) *S* to *N* acyl migration, which are formally the two NCL mechanistic steps, and (iii) desulfurization of the transient thiol moiety to finally afford the ligation at the desired site. The general utility of NCL has been expanded by the development of genetic procedures that introduce thioesters into protein fragments also referred to as expressed protein ligation^88^ (EPL) which efficiently enables access to semi-synthetic proteins bearing particularly difficult sequences. Globally, this ligation–reduction methodology allows ligations at several thiol-free residues such as phenylalanine, alanine, valine, lysine (α- and ε-NH_2_), leucine, glutamine, and proline. Alternatively, the use of thiol-containing auxiliaries (Fig. 5C) allows for a traceless ligation protocol which is particularly relevant for the preparation of glycopeptidic fragments using the so-called sugar-assisted chemical ligation.^89^ This protocol, developed by Wong and co-workers, promotes the ligation at β-GlcNAc-*O*-serine, -*O*-threonine, and -*N*-asparagine residues which represent privileged handles for subsequent (chemo)enzymatic attachment of more complex glycan epitopes for vaccine design. Other ligations with potential as tools for the preparation of glycoprotein vaccines employ ligations at thiol-free residues such as those at phospho-serine and -threonine or at *N*-terminal aspartate and glutamate residues reported by the Payne group (Fig. 5D).^90,91^ Moreover, protocols using Staudinger ligation^92^ (Fig. 5E) and *C*-terminal α-keto acids with *N*-terminal hydroxyl amines (KAHA ligation)^93^ (Fig. 5F) have also gained popularity because they allow complementary ligations to NCL at either natural/unnatural residues or homoserine sites, respectively.^87^ Representative examples of glycoprotein vaccines have been prepared using most of all the aforementioned protocols, from linear solid phase glycopeptide synthesis to more sophisticated NCL-like strategies and include several synthetic antitumour vaccines from mucin glycopeptide antigens as recently reviewed by Kunz and co-workers.^94^

Complementary strategies to the total chemical synthesis of glycoproteins include the aforementioned chemoenzymatic (Fig. 6A) and chemical protein modification protocols (Fig. 6B). Chemoenzymatic protocols,^95^ such as glycoprotein remodelling using glycan processing enzymes as well as glycosylation of apo-proteins, represent attractive approaches to building well-defined homogeneous glycoprotein therapeutics.^84^ The exquisite regio- and stereocontrol achieved with enzymes and the mild and protecting group-free conditions used, makes these transformations compatible with both *in vitro* and *in vivo* conditions. A single glycoform is generated by either homogenizing complex natural glycoforms (glycan trimming) or by introducing human-type enzymes in the natural glycosylation pathways of different eukaryotic cells and bacteria that are able to glycosylate apo-proteins (glycoengineering). This single, naturally occurring *O*- or *N*-linked glycosyl handle can be further elaborated to a more complex but homogeneous epitope using, for instance, endoglycosidases and other glycosyltransferases.

While enzymatic methods rely on *a priori* more sophisticated biotransformations, chemical protein modification protocols^96,97^ represent an attractive and operationally simpler alternative that allow late-stage homogeneous glycoprotein synthesis. By a judicious choice of the residue (natural or unnatural), the reaction conditions, and the chemical transformation, a set of pure glycoproteins with different epitopes and linkers can be prepared. Although a detailed discussion of all synthetic methods available for protein modification is not the topic of the present perspective, representative amino acid tags and linkers are highlighted in Fig. 6 and illustrate the potential of these chemistries to be used for the preparation of synthetically defined glycoprotein vaccines. For a more detailed survey of chemical methods developed for building homogeneous glycoproteins the reader is directed to a number of recent reviews.^98,99^ Relevant examples of such transformations target both natural and unnatural amino acids. Unlike the traditionally used non-specific methods (Fig. 2), recent developments based on site-specific chemical modification of natural residues, such as (i) cysteine and its elimination product (ii) dehydroalanine,^100,101^ allow precise control of the position, type, and number of sugar epitopes attached. For example, these methods proved useful for introducing non-self fluorinated sugars^102^ with potential in vaccine design and imaging using both conjugate thiosugar additions^103^ and S- or Se-exchange reactions.^104,105^ (iii) Tyrosine is another natural residue that can be specifically chemically modified (*e.g.* Tsuji–Trost allylation).^106^ However, the significant progress made in the incorporation of unnatural amino acids into proteins^107^ together with the continuous advent of novel methodologies for their modification (*e.g.* metal-mediated strategies)^108^ has opened up new opportunities to explore and exploit bioorthogonal transformations^109^ using these reactive handles. Among the new unnatural residues, some of the more representative examples include (iv) homoallylglycine (Hag) and other alkene tags for thiol–ene reactions,^110^ (v) *S*-allylcysteine (Sac) or *Se*-allylselenocysteine (Seac) for cross-metathesis reactions,^111^ (vi) carbonyl handles, typically employed in oxime ligations,^112^ and (vii) azidohomoalanine (Aha) and other azide-moieties, which represent an attractive way of introducing glycans *via* well-known, robust transformations such as Cu(i)-catalyzed azide alkyne cycloadditions (CuAAC),^113^ Staudinger ligations,^114^ traceless Staudinger,^115^ and more recently Cu-free cycloaddition protocols with strained alkynes.^116^ Other important transformations include those using (viii) homopropargylglycine (Hpg) and other alkyne handles for CuAAC, thiol–yne reactions,^117^ and Sonogashira cross-couplings as well as (ix) Suzuki cross-couplings with *p*-halophenylalanine tags.^118^ When combined with a chemoenzymatic approach that uses endoglycosidases, proteins chemically modified with monosaccharides can be efficiently elaborated to more complex glycoproteins,^119^ which represents a step forward in the preparation of complex glycoproteins with a minimum of modification steps.

Each of the methods described in this section has the potential to become an important tool to efficiently prepare and study proteins with defined glycosylation patterns as vaccine candidates.

## 5 Conclusions

Glycoproteins are the centre of many vaccines that are either in the clinic or in advanced clinical trials.^1^ Typically, for a specific and robust immune response the carbohydrate antigen must be conjugated to a protein carrier. This process often results in heterogeneous products, containing a mixture of species with different antigen to protein ratios at different sites and, potentially, different pharmacokinetic and immunological properties. Oligosaccharide synthesis and site-specific protein modification methodologies have seen immense advances over the past decade and are now available. With these, it is now possible to design and synthesize chemically defined glycoconjugate vaccines with varying carbohydrate densities, conformations, and shapes, which would rely on an easier physicochemical characterization in comparison to current carbohydrate-based vaccines, and hopefully lead to the selection of candidates with enhanced safety and efficacy. In addition, it has been recently shown that T-cells can recognize the carbohydrate portion of the glycopeptide presented to them by MHCII.^5^ We anticipate that the use of pure glycoprotein vaccine constructs with epitopes displayed at precise sites and forming defined shapes within the protein carrier will help in understanding the antigen presentation mechanisms and result in a robust immune response.
